# Epithelial and Stromal Characteristics of Primary Tumors Predict the Bone Metastatic Subtype of Prostate Cancer and Patient Survival after Androgen-Deprivation Therapy

**DOI:** 10.3390/cancers14215195

**Published:** 2022-10-23

**Authors:** Pernilla Wikström, Sofia Halin Bergström, Andreas Josefsson, Julius Semenas, Annika Nordstrand, Elin Thysell, Sead Crnalic, Anders Widmark, Camilla Thellenberg Karlsson, Anders Bergh

**Affiliations:** 1 Department of Medical Biosciences, Pathology, Umeå University, 901 87 Umeå, Sweden; 2 Department of Surgical and Perioperative Sciences, Urology and Orthopedics, Umeå University, 901 87 Umeå, Sweden; 3 Wallenberg Centre for Molecular Medicine, Umeå University, 901 87 Umeå, Sweden; 4 Department of Radiation Sciences, Oncology, Umeå University, 901 87 Umeå, Sweden

**Keywords:** prostate cancer, bone metastases, metastatic subtypes, Ki67, PSA, androgen receptor, smooth muscle actin, PDGFRB, SDF1, ERG

## Abstract

**Simple Summary:**

Metastatic prostate cancer is a lethal disease and metastasis-specific treatments need to be developed. Mechanisms driving metastases and primary tumor growth could be different, but this is largely unexplored. We previously discovered that bone metastases can be separated into transcriptomic-based subtypes, showing different responses to standard androgen-deprivation therapy for metastatic prostate cancer. One subtype, named MetB, is particularly aggressive and has the worst prognosis. Here, we describe similarities and differences between primary tumors and their metastases, and specifically examine if the development of specific subtype of bone metastases can be predicted by analyzing the primary tumor. Results show that many aspects of prostate cancer bone metastases morphology are related to those in the primary tumor, while others are not. Importantly, men with primary tumors with high cell proliferation and low cellular PSA expression tend to develop metastases enriched for the MetB subtype, have poor prognosis, and need complementary treatment to standard hormone treatment.

**Abstract:**

Prostate cancer (PC) bone metastases can be divided into transcriptomic subtypes, by us termed MetA-C. The MetB subtype, constituting about 20% of the cases, is characterized by high cell cycle activity, low androgen receptor (AR) activity, and a limited response to standard androgen deprivation therapy (ADT). Complementary treatments should preferably be introduced early on if the risk of developing metastases of the MetB subtype is predicted to behigh. In this study, we therefore examined if the bone metastatic subtype and patient outcome after ADT could be predicted by immunohistochemical analysis of epithelial and stromal cell markers in primary tumor biopsies obtained at diagnosis (*n* = 98). In this advanced patient group, primary tumor International Society of Urological Pathology (ISUP) grade was not associated with outcome or metastasis subtype. In contrast, high tumor cell Ki67 labeling (proliferation) in combination with low tumor cell immunoreactivity for PSA, and a low fraction of AR positive stroma cells in the primary tumors were prognostic for poor survival after ADT. Accordingly, the same tissue markers were associated with developing metastases enriched for the aggressive MetB subtype. The development of the contrasting MetA subtype, showing the best response to ADT, could be predicted by the opposite staining pattern. We conclude that outcome after ADT and metastasis subtype can, at least to some extent, be predicted by analysis of primary tumor characteristics, such as tumor cell proliferation and PSA expression, and AR expression in stromal cells.

## 1. Introduction

Cancers confined to the prostate can be cured by surgery or by radiotherapy, but when the malignant cells form clinically detectable bone metastases, the disease is lethal. Current therapies for metastatic prostate cancer (PC) primarily target androgen synthesis and/or androgen receptor (AR) signaling (called androgen deprivation therapy, ADT). Often cytotoxic drugs such as taxanes are also given. This offers temporary relief, but eventually treatment resistance develops. The duration of the initially favorable response is highly variable between patients. We have shown that human PC bone metastases can be separated into different subgroups with different transcriptome, proteome, metabolome, genetics, epigenetics, immune phenotypes, clinical behavior, and response to standard treatments [1,2,3,4,5,6,7,8]. Or in other words, there are different types of aggressive PC. The most common transcriptomic subtype (approximately 70–80% of cases), by us named MetA, is characterized by high expression of androgen-regulated genes (including the prostate specific antigen, PSA), relatively low cell proliferation, a luminal cell phenotype, and a relatively favorable response to ADT. The most aggressive metastasis subtype (approximately 10–20% of cases), named MetB, is characterized by high cell proliferation, a dedifferentiated luminal cell phenotype with low PSA secretion, and the poorest response to ADT [4,5]. The smallest subtype (up to approximately 10% of cases), named MetC, is characterized by high expression of stroma-related genes, high immune cell response, and an intermediate to good ADT response [4,5]. The diverse gene expression patterns in MetA-C indicate that their clinical behaviors are driven by different molecular mechanism and that they thereby should be treated differently [4,5].

As MetB metastases show a limited response to standard ADT, additional treatments should probably be introduced as early as possible, if the risk of developing such metastases is determined high. Markers for the subsequent development of MetB metastases should ideally be detectable already in the primary tumor, and not reliant on analysis of metastases samples. Whether this is possible is, however, not known. The global gene expression pattern and proteome are highly different in primary tumors vs. bone metastases [2,5], and the response to ADT is markedly dependent on the tumor microenvironment [9]. Discordancy in molecular subtype between the primary tumor and its metastases is common in other types of cancer, for example in breast and colon cancer [10,11], suggesting large molecular differences between primary tumors and their metastases. On the other hand, we showed in the Thysell et al. 2019 paper that metastases with relatively high immunoreactivity of the marker for cell proliferation Ki67 and relatively low immunoreactivity for PSA were enriched for the MetB subtype [5]. Interestingly, the matched primary tumors of MetB cases were generally characterized by relatively high Ki67 and low PSA in primary tumor epithelial cells, and a reactive stroma response with reduced AR-levels in the primary tumor stroma [5]. In addition, primary tumors with relatively high Ki67 and low PSA responded poorly to ADT [5,12], and so did cases with low AR levels in their stroma [13]. Therefore, the aim of this study was to explore if the metastatic subtype and patient prognosis after ADT could be predicted by analyzing morphological characteristics of the primary tumor.

## 2. Materials and Methods

### 2.1. Patients

The study includes 98 patients with metastatic PC from whom paired samples of metastatic and primary tumor tissue were available (Table 1). In most cases (*n* = 90), the metastatic tissue was obtained during surgery due to metastatic spinal cord compression or pathological fractions at Umeå University Hospital between 2003–2020. In other cases, core biopsies were obtained during treatment for castration-resistant PC prostate cancer (CRPC) from the iliac crest (*n* = 7), or rib (*n* = 1) metastases. At the time of sampling, patients were either hormone-naïve (*n* = 13), castration-resistant (*n* = 81), or treated with ADT for a shorter period ranging between 1 to 17 days (short-term castrated, *n* = 4). Diagnostic prostate biopsies were retrospectively retrieved from the pathology units at the hospitals in Luleå, Umeå, Sundsvall, and Östersund. All patients gave their informed consent, and the study was conducted in accordance with the Declaration of Helsinki. The study was approved by the regional ethic review boards in Umeå (Dnr 03-158, Dnr 04-26M, 24 August 2007).

### 2.2. Morphology

Formalin-fixed paraffin-embedded (FFPE) samples of paired primary and metastasis prostate tumors from 98 patients were available. The tissue samples were immune-stained for the epithelial and stroma cell markers Ki67, PSA, AR, ETS-related gene (ERG), and platelet-derived growth factor receptor β (PDGFRβ), as earlier described [5,14,15,16]. Sections were also stained for smooth muscle actin (SMA) (M0851/clone 1A4, DAKO Agilent, Santa Clara, CA, US, 1:150) using the automated BenchMark ULTRA system (Roche Diagnostics, Indianapolis, IN, US) with Cell Conditioning 1 (CC1) as antigen retrieval buffer and developed using the Ultraview Universal DAB Detection kit (Ventata Systems, Tuscon, Arizona, US). Stroma derived factor 1 (SDF1) (MAB350/clone 79018, R&D Systems, Minneapolis, MN, US) 1:50 overnight) was stained manually using Tris-EDTA pH9 as antigen retrieval and developed with DAB (Mach3Mouse HRP-Polymer Detection, M3M530, Biocare Medical, Pacheco, CA, US). The staining for AR, Ki67, PSA, and ERG in epithelial cells were scored as earlier described [5,14]. Briefly, the fraction (%) of Ki67+ cells were calculated, and epithelial ERG was scored as positive or negative. For AR and PSA, H-scores were calculated, ranging from 0–12, based on fraction of stained cells (0–4) and staining intensity (0–3). The fraction of stroma cells positive for AR and for Ki67 was calculated after examining at least 500 stroma cells per sample. The volume density of ERG+ endothelial cells, SDF-1+ stroma, SMA+ stroma, and PDGFRβ+ stroma was quantified using a square-lattice mounted in the eyepiece of the microscope counting grid-intersection falling on the stained tissue component and on reference space. The morphological data of bone metastases and their relations to the transcriptomic subtypes MetA-C (see below) and patient prognosis after ADT have, to some extent, been described and are here complemented with additional data and cases, and then related to morphological data of the primary tumors [4,5].

### 2.3. Classification of the Metastasis Subtypes MetA-C

For 70 of the metastasis cases, frozen tissue samples had been previously profiled by whole-genome transcriptomic analysis [4,5]. The fractions of MetA-C (0.0–1.0) in each metastasis sample were determined based on the expression levels of 157 MetA-C-associated genes. Samples were classified based on their dominant MetA-C fraction, as previously described [4].

### 2.4. Statistics

Continuous variables were given as median (25th; 75th percentiles) and ordinal variables were given as number (percentage). Non-parametric statistics were used to compare groups and for correlation analyses (the Mann-Whitney *U* test, the Spearman rank correlation test, and the Chi-squared test). Cancer-specific survival was analyzed using the log-rank test with death from PC as event and other causes as censored events. Follow-up started at the date of ADT and ended at the date of death or the latest follow-up. Multivariate Cox proportional hazard models were built to evaluate relationships between different prognostic variables, adjusted for patient age, serum PSA, and tumor differentiation (ISUP grade). All tests were two sided and *p*-value less than 0.05 were considered statistically significant. Statistical analysis was performed using the IBM SPSS Statistics 28.0.1 software.

## 3. Results

### 3.1. Epithelial and Stroma Cell Markers in paired Primary Tumors and Metastases

All but 3 patients in this cohort, where all eventually developed bone metastases, were diagnosed with high grade tumors (ISUP grade 3–5) (Table 1). In the primary tumors, the epithelial cells were growing in a stroma containing smooth muscle cells, cancer associated fibroblasts (CAFs), blood vessels, different types of immune cells, and nerves as reviewed [17,18]. The amount of stroma and its composition in primary tumors varied between patients. Tumor cell histology in bone metastases resembled that in their primary tumors, although the fraction of tumor areas with glandular differentiation was lower in the metastases compared to the primary tumors.

**Figure 1:**
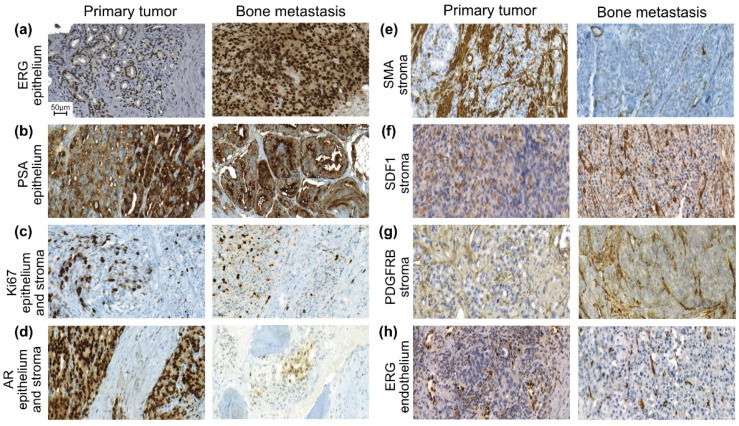
Sections from paired primary prostate tumors and bone metastases immune stained for ETS-related gene (ERG) (**a**,**h**), prostate specific antigen (PSA) (**b**), the marker for proliferation Ki67 (**c**), androgen receptor (AR) (**d**), smooth muscle actin (SMA) (**e**), stroma derived factor 1 (SDF1) (**f**), and platelet-derived growth factor receptor β (PDGFRB) (**g**), all seen at the same magnification (see scalebar in (**a**)). (**a**) ERG positive tumor cells in a primary tumor and in the paired metastasis. (**b**) Moderate (left side) to high (right side with glands) PSA staining in a primary tumor. Moderate PSA staining and glandular differentiation was seen in the metastasis (classified as MetA). (**c**) Sections with relatively high Ki67 labeling in epithelium and stroma in a primary tumor and in the paired bone metastasis. (**d**) AR positive epithelial and stroma cells were seen in the primary tumor. In the metastasis (classified as MetA), a nest of AR positive tumor cells was growing in a bone marrow space and a few AR positive cells are seen on the bone surface. (**e**) Numerous SMA positive smooth muscle cells are seen in the primary tumor, whereas only few cells were SMA positive in the paired bone metastasis (here only in blood vessel walls). This metastasis, lacking bone structures, was classified as MetB. (**f**) SDF-1 positive cells were seen in the stroma in the primary tumor, mainly in blood vessel walls. Such cells were also seen in the bone metastasis stroma. (**g**) PDGFRB positive stroma cells, mainly in blood vessel walls and in fibroblasts, were seen in the primary tumor stroma and in the paired metastasis (classified as MetB). (**h**) ERG positive endothelial cell nuclei were seen in the primary tumor and in its bone metastasis. Note that several tumor cells are lying close to endothelial cells. In this case, the tumor cells were ERG negative. This metastasis was classified as Ki67 high/PSA low but with unknown MetA-C status. Blood vessels with ERG positive endothelial cells and walls positive for SMA, PDGFR-beta, and SDF-1 were a dominant component of the bone metastasis stroma.

The tumor cell microenvironment in the bone metastases was strikingly different from that in the primary tumors, and it also varied between cases. In some patients, the metastatic cells were growing within bone marrow spaces containing few fibroblast-like cells, but in close contact with bone surface, blood vessels, blood forming bone marrow cells, and adipocytes, (Figure 1). In other cases, little bone and bone marrow remained and the tumor epithelial cells were growing in a blood vessel-rich stroma containing some CAFs and inflammatory cells (Figure 1).

#### 3.1.1. Tumor Epithelial Cell Markers

Primary tumors and metastases were immune-stained for ERG (Figure 1), a marker for the TMPRSS2-ERG fusion gene [16], and markers for proliferation (Ki67), differentiation (PSA), and hormone receptors (AR).

Thirty-seven percent (37%) of the analyzed primary tumors (32/86) were ERG+ (defined as positive if any part of the tumor was positive), while only 27% (25/92) of the metastases were ERG+. All metastases showed homogeneous ERG staining. In the primary tumor biopsies, 6 cases contained both ERG+ and ERG− areas. The ERG status was highly correlated between primary tumors and their metastases (Table 2).

The primary tumor and metastasis tissue showed variable immune-staining of Ki67, PSA, and AR in tumor epithelial cells both within and among cases (Figure 1). The tumor cell proliferation (fraction of Ki67+ cells), PSA staining score, and AR staining score in the primary tumors were correlated to those of paired metastases samples from the same patient (Table 2). Overall, however, the median tumor cell proliferation was significantly lower in primary tumors than in metastases, while the median PSA and AR staining scores were significantly higher (Table 2).

#### 3.1.2. Stromal Cell Markers

We also examined the following markers in the stroma of primary tumors and metastases; Ki67, AR, smooth muscle actin (SMA), stroma-derived factor 1 (SDF-1), PDGFRβ, and ERG as a marker of endothelial cells.

Proliferating (Ki67+) stroma cells were rare in the primary tumors, but significantly more abundant in the corresponding metastases (Figure 1, Table 2). In bone metastases, the Ki67+ stroma cells were mainly endothelial or mural vascular cells, and some CAF-like cells (Figure 1). The fraction of Ki67+ stroma cells in primary tumors was not correlated to that of paired metastases (Table 2).

AR+ stroma cells, particularly AR+ smooth muscle cells, were common in the primary tumors although a large variability could be seen between different cases (Figure 1). In metastases, AR staining was pronounced in the nucleus of metastatic tumor epithelial cells, whereas most cells in the metastasis stroma were AR negative (Figure 1). A detailed examination showed weak to moderate nuclear AR staining in few osteoblasts, endothelial cells, and CAF-like cells, as well as in the subsets of inflammatory cells (Figure 1). The fraction of AR+ stroma cells was significantly higher in primary tumors than in the bone metastases (Table 2), despite a weak correlation seen between AR+ stroma cells in primary tumors and paired metastasis samples (Table 2).

In primary tumors, SMA+ smooth muscle cells (generally AR+), SMA+ fibroblasts (CAFs) and SMA+ cells in blood vessel walls were all common and constituted almost 1/5 of the tumor volume, while in the bone metastases, SMA+ cells were significantly less abundant (Figure 1) (Table 2). Most SMA+ cells in metastases were seen in blood vessel walls (pericytes and vascular smooth muscle cells, generally AR-), whereas SMA+ fibroblast (CAF)-like cells were less common (Figure 1). The volume density of SMA+ cells in primary tumor was not correlated to that of the paired metastases (Table 2).

Moreover, staining of the chemokine SDF-1 was seen in fibroblasts-like cells and in blood vessel walls, and in some cases also in tumor epithelial cells in the primary tumors (Figure 1). In bone metastases, SDF-1 staining was observed in thin-walled blood vessels adjacent to tumor epithelial cell nests and in some fibroblast-like cells in the stroma (Figure 1). In some patients also metastatic tumor cells stained positively for SDF-1. The volume density of SDF-1+ stroma cells was significantly lower in primary tumors than in metastases (Table 2).

Moreover, PDGFRβ was abundantly expressed in the primary tumors, mainly in pericytes and CAFs located at the epithelial-stromal interphase (Figure 1). In bone metastases, vascular mural cells and CAFs (when present), particularly at the epithelial-stromal interface, were positive for PDGFRβ (Figure 1). The volume density of PDGFRβ+ stroma cells was slightly higher in metastases than in the primary tumors, with no clear correlation between the two (Table 2).

Endothelial cells were ERG+ and, therefore, ERG was used to examine blood vessel density. Many tumor cells, both in primary tumors and in metastases, were growing in close proximity to ERG+ endothelial cells and formed a morphological foundation for angiocrine interactions (Figure 1). The volume density of ERG+ endothelial cells was similar in primary tumors and in corresponding metastases (Figure 1) (Table 2).

#### 3.1.3. Morphological Similarities between Paired Primary Tumors and Metastases Diverse with Time

In this patient cohort, the timespan between sampling of the primary tumor biopsies and the paired bone metastasis samples ranged from 0 to 17 years, with a median time of 1.9 years (Table 1). To explore if longer time between sampling made primary tumors and metastases less morphologically similar, and vice versa, cases were analyzed in two groups based on time between sampling (above or below median). As anticipated, stronger correlation values were observed between the immunohistochemical markers in paired primary tumor and metastases when analyzed in samples collected within a short compared to a long time-span (Table S1 vs. Table S2).

### 3.2. Epithelial and Stromal Markers in Primary Tumors, in Relation to Patient Outcome and to Metastasis Subtypes

We then analyzed if any of the epithelial or stroma markers analyzed in primary tumors were related to patient survival after ADT or to the metastasis morphology and transcriptomic subtype, MetA-C [4].

#### 3.2.1. Tumor Epithelial and Stromal Markers in Primary Tumors Predict Prognosis

**Figure 2:**
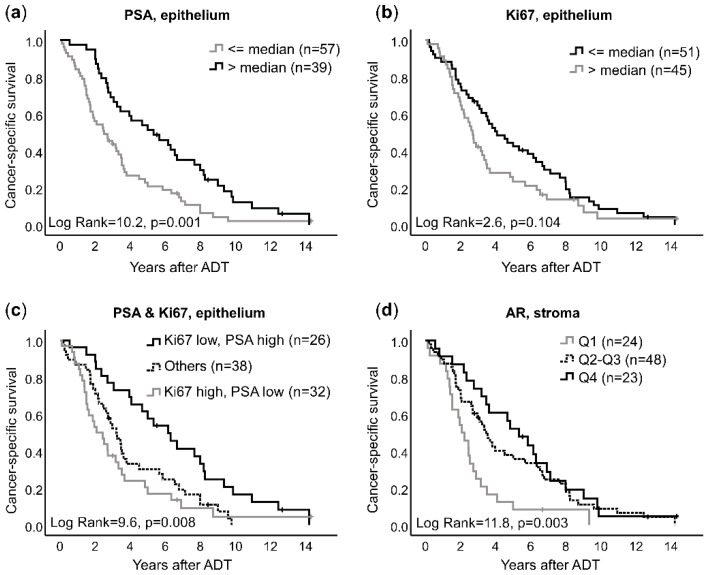
Tumor cell proliferation (Ki67) and expression of prostate-specific antigen (PSA) as well as stromal cell expression of the androgen receptor (AR) in prostate tumor biopsies, in relation to patient survival after treatment with androgen-deprivation therapy (ADT). Tumors (*n* = 98) were dichotomized by immunoreactivity scores for (**a**) PSA at the median cut-off level (h-score of 8), (**b**) Ki67 (proliferation) at the median cut-off level (13%), (**c**) A combinatory score using the same cut-off levels as in a and b stratifying tumors into 4 groups; “Ki67 low, PSA high”, “Ki67 high, PSA high”, “Ki67 low, PSA low”, and “Ki67 high, PSA low”, of which the two middle groups were analyzed together as “Others”, and (**d**) AR in stromal cells at quartile levels (Q1 ≤ 10%, Q2–Q3: 10–27%, Q4 > 27%). The dichotomized tumor groups were analyzed in relation to cancer-specific survival after ADT, according to Kaplan-Meier survival analysis.

Of the epithelial markers examined, a reduced PSA immunoreactivity in tumor epithelial cells of diagnostic prostate biopsies was associated with short patient survival after ADT (Figure 2a), while the Ki67 labeling index was not significantly associated with survival in this patient cohort (Figure 2b). However, by stratifying patients based on the median immunoreactivity levels for both Ki67 and PSA, groups of patients with particularly favorable (low Ki67, high PSA) or poor (high Ki67, low PSA) prognosis were identified, while the others showed an intermediate prognosis (median survival times 6.2, 2.1, and 3.2 yrs., respectively, *p* = 0.0081) (Figure 2c).

Of the stroma cell markers, low density of AR+ stroma cells in primary tumors were associated with short survival after ADT (Figure 2d).

The classification of patients into groups based on Ki67 and PSA immunoreactivity (the combinatory Ki67, PSA score) together with fraction of AR+ stroma cells added independent prognostic information to serum PSA at diagnosis (Table 3). Notably, the primary tumor ISUP grade was not related to survival after ADT in this patient cohort (Table 3). The densities of SDF1+, SMA+, Ki67+, PDGFRβ+ stroma cells and ERG+ endothelial cells in the primary tumors were not obviously associated with patient prognosis.

#### 3.2.2. Tumor Epithelial and Stromal Markers of Primary Tumors Associated with the Metastasis Morphology and Transcriptomic Subtype

Metastases corresponding to primary tumors with “Ki67 high, PSA low” immunoreactivity showed significantly higher cell proliferation in both the epithelial and stromal fractions, a lower PSA immunoreactivity and a higher density of endothelial cells compared to metastases corresponding to primary tumors with “Ki67 low, PSA high” immunoreactivity (Table 4), indicating that the Ki67 and PSA expression in primary tumors is associated with the morphological subtype of bone metastases.

A majority of the metastatic samples (*n* = 70) had been previously characterized for their transcriptomic subtype (MetA-C) (4,5). From the expression levels of 157 MetA-C associated genes, the tumor fraction (%) corresponding to the MetA, B and C phenotype, respectively, were estimated for each metastasis sample (4). Based on these estimations, metastases corresponding to “Ki67 high, PSA low” primary tumors contained a significantly lower fraction of the MetA and a significantly higher fraction of the MetB subtype compared to “Ki67 low, PSA high” primary tumors (Table 4). Importantly, if classified based on their dominating MetA-C fraction, no metastases of the aggressive MetB subtype were found among patients with “Ki67 low, PSA high” primary tumors (Table 5). All MetB metastases were instead found among patients with “Ki67 high” and/or “PSA low” primary tumors (Table 5). As single variables, Ki67 in primary tumor epithelium showed a weak but significant correlation to the fraction of MetB and a moderate inverse correlation to the fraction of MetA (Table S3). In contrast, PSA score in primary tumors moderately correlated to the fraction of MetA in the metastases (Table S3).

Weak correlations were also observed between the fraction of AR+ stromal cells in the primary tumor and the fractions of MetA (positive correlation) and MetB (inverse correlation) in the metastases (Table S3). The fraction of proliferating stromal cells was positively correlated to the fraction of MetB cells (Table S3).

Serum PSA at primary diagnosis was positively related to the PSA staining score of metastases and the fraction of MetA, and inversely related to the tumor cell proliferation (Ki67) in the metastases and the fraction of MetB (Table S3). The ISUP grade of the primary tumors showed no correlation to the Ki67 or PSA immunostaining in the metastases nor to the transcriptomic subtypes, MetA-C (Table S3). For further details about correlations between the examined markers, please see Table S3.

## 4. Discussion

By thorough characterization of human PC bone metastatic tissue, we have previously discovered that metastatic PC can be separated into subgroups with different biology and different responses to standard ADT. The most aggressive bone metastases can be recognized by either: (1) analysis of the global transcriptome to identify cases of a subtype termed MetB [4,5], (2) analysis of the global proteome to detect a subtype termed BM2 [2], or (3) immunostaining to identify cases with particularly high Ki67 and low PSA [5]. In other words, these three methods might be used to identify patients in high need of more effective treatment given as an early complement to ADT. However, these methods require that bone metastases are biopsied and analyzed. The current study was therefore conducted to explore if it is possible to predict the molecular and histological subtypes of metastases by instead examining the primary tumor. If so, subtype-specific metastasis treatments could be initiated at the earliest possible time-point, i.e., at diagnosis. 

Taken together, our main results indicate that patients with primary tumors showing low tumor cell proliferation in combination with high PSA expression (“Ki67 low, PSA high”) will develop metastases primarily of the MetA subtype that will show a favorable response to standard ADT. In contrast, patients with primary tumors with high proliferation and/or low PSA expression will develop metastases enriched for the MetB subtype that will show a poor response to ADT, and they are thus in need of early complementary therapies. However, it is important to note that our conclusion suggesting that the metastatic phenotype and patient prognosis after ADT can be predicted by examining specific characteristic of the diagnostic primary tumor biopsies applies to patients similar to the ones examined here, i.e., to patients with already established metastatic disease at diagnosis, where the serum PSA, ISUP grades, and Ki67 labeling index are higher than in the average patient diagnosed with PC [12](see also www.npcr.se, accessed on 1 September 2021). How this conclusion applies to patients diagnosed at earlier disease stage remains to be explored.

In the current study, we did not have access to necessary amount of tissue to perform analysis of the global transcriptome or proteome in the primary tumor biopsies. Therefore, our analysis was restricted to microscopic evaluation. Importantly, neither metastasis subtype nor the response to ADT were related to primary tumor ISUP grade (ranging from 2–5). In line with our previous hypothesis, based on 52 paired cases [5], the primary tumors of patients who developed the most aggressive and ADT-resistant metastases were characterized by particularly high cell proliferation and reduced cellular PSA (marker for low AR activity and cellular dedifferentiation) in the tumor cells. In line with this, a gene-expression pattern in primary tumors, related to high cell proliferation and cell dedifferentiation, the Decipher test, may also be used to identify patients that later responded poorly to ADT [19,20].

The aggressive “high Ki67, low PSA” primary tumors were characterized by a reactive stroma response with increased cell proliferation and reduced fraction of AR+ stromal cells compared to other tumors. Their established bone metastases were enriched for the MetB subtype forming a proliferating and largely AR negative stroma with high vessel density. Metastases from the most contrasting, less aggressive phenotype (“low Ki67, high PSA”) instead grew in a stroma where blood vessels and proliferating cells were less abundant, and they were enriched for the MetA subtype. These cases demonstrated less signs of a reactive stroma in their primary tumors, which may suggest that tumor cells of a particular subtype may secrete factors, inducing a related stroma response in primary tumors and in metastases. In the normal prostate and in primary PC, epithelial cell proliferation and differentiation are known to be controlled by bidirectional stroma-epithelial cell interactions, driven by androgen-action in AR+ epithelial and AR+ stromal cells [17,18,21]. Accordingly, tumor cell characteristics, such as Ki67 and PSA staining scores were correlated to several stromal markers in both primary tumors and in metastases. One factor associated with poor response to ADT, as earlier suggested [13], was low AR levels in the stroma of primary tumors. If AR-downmodulation in the stroma drives proliferation and dedifferentiation of tumor epithelial cells or if dedifferentiated and proliferating epithelial cells lack the ability to maintain a differentiated AR+ stroma smooth muscle cell phenotype remains to be explored.

Interestingly, from a set of stromal markers (PDGFRβ, SMA, and SDF1) previously related to primary tumor aggressiveness [15,22,23,24], none appeared to be associated with patient prognosis after ADT in this patient cohort. Furthermore, they were also not predictive of the metastasis subtypes. Possibly, some prognostic markers may be useful for separating low-risk vs. high-risk tumors at diagnosis, while being of limited use for separating different types of metastatic cancers. The reasons for this remain to be examined.

Although our current observations suggest that the morphology of primary tumors to some extent is related to the metastasis phenotypes, it should be noted that the correlation coefficients for individual factors measured in primary tumors and paired metastases samples were all relatively low. The weak correlations may be related to the fact that the primary tumors were all untreated, whereas the metastases were mainly collected at later time-points, some-times several years after the primary tumor, generally after ADT and sometimes also after additional treatments for castration-resistance. This would have allowed for clonal expansion over time. In addition, PC is multifocal and polyclonal, and the diagnostic biopsies may have failed to sample the tumor area forming the metastases. Furthermore, quantification of “hot spots” (potential origin of metastatic clones) instead of calculating average marker values for the diagnostic biopsies could result in higher correlations. It is also likely that some key aspects of the metastasis phenotypes are unrelated to the primary tumor characteristics, for which the bone microenvironment instead could be central for determining the metastasis phenotype and its response to treatment. For example, when the same AR+ PC cell line was implanted in the prostate and in the bone, tumor response to castration therapy was prominent in the prostate but largely absent in the bone, pointing out the importance of site-specific microenvironments for therapy response [9].

By categorizing the different markers compared in primary tumor biopsies and paired metastasis samples into different groups we suggest that: (1) markers for genetic alterations, such as the TRMPSS2-ERG fusion gene [16], are highly correlated and largely maintained over time, (2) markers for the epithelial cell phenotype, such as proliferation and differentiation are moderately correlated, probably influenced by the tumor cell genome, bidirectional signaling to and from the tumor microenvironment, and by treatments and time, and (3) stromal markers are less correlated. Consequently, if metastasis characteristics and responses to treatments are to be determined by genetic drivers, analysis of the primary tumor is of prime importance, but if the therapy response is governed largely by the bone microenvironment, analysis of the primary tumor may be insufficient. Additional studies are needed to clarify this.

## 5. Conclusions

In conclusion, in metastatic PC patients, selected primary tumor characteristics such as high tumor cell proliferation (Ki67 index) in combination with low PSA expression, and a reactive stroma response with loss of AR+ stromal cells and increased stromal cell proliferation, can predict the development of metastases of a particularly aggressive subtype in high need of complementary treatment to standard ADT. Further studies are needed to verify those findings and to explore if treatment stratification of patients into complementary therapies to ADT based on those primary tumor characteristics will improve patient survival.

## Figures and Tables

**Table 1:** Clinical characteristics of 98 patients with bone metastatic prostate cancer from whom paired metastatic and primary tumor tissue were available.

	Median (25th; 75th Percentiles)
Age diagnosis (yrs.)	69 (65; 75)
Age metastasis surgery (yrs.)	73 (68; 78)
Serum PSA diagnosis (ng/mL)	78 (30; 440)
Serum PSA metastasis surgery (ng/mL)	140 (32; 470)
Time btw sampling of primary tumor and metastasis tissue biopsies (yrs.)	1.9 (0.75; 4.6)
**ISUP grade at diagnosis:**	**N**
2	3 (3%)
3	31 (32%)
4	34 (35%)
5	30 (31%)
**Castration therapy ^a^:**	**N**
None (hormone-naïve)	13 (13%)
Short-term ^b^	4 (4%)
CRPC ^c^	81 (83%)
**Treatment for CRPC:**	**N**
Bicalutamide	46 (49%)
Chemotherapy	20 (20%)
Abiraterone acetate	10 (10%)
Enzalutamide	4 (4%)
Ra223	3 (3%)
Zoledronic acid/Denosumab	2 (2%)

Continuous variables given as median (25th; 75th percentiles). ^a^ Castration therapies given prior to collection of metastasis tissue samples included surgical ablation, LHRH/GnRH agonist therapy or bicalutamide treatment. ^b^ Castration therapy for 1–17 days before metastasis tissue sampling. ^c^ Defined to have castration-resistant prostate cancer (CRPC) due to disease progression during castration therapy. PSA, prostate specific antigen; ISUP, International Society of Urological Pathology.

**Table 2:** Immunoreactivity of selected epithelial and stromal markers of paired primary tumor and metastasis biopsies, sampled with a median time of 1.9 years in-between.

	Primary Tumor	Metastases	R_S_ (n)
**Epithelial markers**			
ERG (pos./total) Epithelial markers	30/80	21/80 **	0.71 ***
Ki67 (%), *n* = 98	13 (7.5; 20)	16 (9.3; 25) *	0.38 ***
PSA (score), *n* = 98	8 (6; 12)	6 (4; 9) *	0.37 ***
AR (score), *n* = 92	12 (8; 12)	8 (4; 12) ***	0.22 *
**Stromal markers**			
Ki67 (%), *n* = 75	1.5 (0.95; 2.9)	5.0 (3.0; 7.0) ***	0.074
AR (%), *n* = 68	19 (13; 28)	2.7 (1.5; 4.0) ***	0.27 *
SMA density (%), *n* = 50	16 (12; 18)	5.3 (3.5; 8.3) ***	0.097
SDF-1 density (%), *n* = 51	3.1 (2.1; 4.1)	4.3 (3.4; 6.1) ***	−0.27
PDGFRβ density (%), *n* = 37	9.1 (5.0; 13)	10 (8.3; 13) *	0.26
ERG, endothelium density (%), *n* = 69	1.0 (0.66; 1.5)	1.0 (0.62; 1.4)	0.44

Marker values are median and 25th and 75th percentiles, * *p* < 0.05, ** *p* < 0.01, *** *p* < 0.001. ERG, ETS-related gene; Ki67, marker for proliferation; PSA, prostate specific antigen; AR, androgen receptor; SMA, smooth muscle actin; SDF-1, stroma derived factor 1; PDGFRβ, platelet-derived growth factor receptor β.

**Table 3:** Multiple Cox regression analysis of specified immunoreactivity scores in primary tumor biopsies, in relation to cancer-specific survival after androgen-deprivation therapy (ADT).

Clinical Variables	HR (95% CI)	P
Age at diagnosis (yrs.)	1.0 (1.0–1.1)	0.066
Serum PSA at diagnosis (ng/mL)	1.1 (1.0–1.2)	0.026
ISUP grade at diagnosis	1.0 (0.78–1.4)	0.78
**Combinatory Ki67, PSA score ^a^**		
Low Ki67, high PSA, *n* = 26	ref.	
Others, *n* = 35	2.9 (1.6–5.3)	0.00028
High Ki67, low PSA, *n* = 31	2.5 (1.3–4.8)	0.0065
**AR in stroma cells ^b^**		
Q1, *n* = 23	ref.	
Q2–3, *n* = 47	0.46 (0.25–0.86)	0.014
Q4, *n* = 22	0.37 (0.18–0.74)	0.0050

^a^ A combinatory immunoreactivity score for the marker of proliferation Ki67 and prostate specific antigen (PSA) constructed based on the median values (13% and 8%, respectively) stratified primary tumors into 3 groups: “low Ki67, high PSA”, “high Ki67, low PSA”, and “others”. ^b^ The androgen receptor (AR) score dichotomized based on quartiles (Q1, Q2–3, Q4; cut-offs 10 and 27%).

**Table 4:** Primary tumors differentiated based on combined immunostaining of the marker for proliferation Ki67 and prostate specific antigen (PSA), in relation to clinical variables, epithelial and stromal markers, and the transcriptomic metastasis subtypes MetA-C.

	Ki67 Low, PSA High ^a^	Ki67 High, PSA Low ^a^	Others ^a^
	*n* = 26	*n* = 34	*n* = 38
Age at diagnosis (yrs.), *n* = 97	71 (65; 78)	70 (65; 76)	68 (64; 73)
Serum PSA at diagnosis (ng/mL), *n* = 96	110 (41; 940)	44 (26; 96)	97 (49; 590)
ISUP, *n* = 98	4 (3; 5)	4 (4; 5)	4 (3; 4)
**Primary tumors**			
Ki67 epithelium (%), *n* = 98	7.5 (6; 10)	22 (18; 37) ***	12 (7.0; 16) *
PSA epithelium (score), *n* = 98	12 (9; 12)	6 (3; 7) ***	8 (6; 10) ***
AR epithelium (score), *n* = 97	12 (8; 12)	12 (8; 12)	12 (9; 12)
ERG epithelium (pos./total), *n* = 86	5/22	11/30	16/34
Ki67 stroma (%), *n* = 95	1 (0.5; 1.5)	2.8 (1.1; 4.0) ***	1.2 (0.63; 2.0)
AR stroma (%), *n* = 97	23 (16; 34)	9.5 (6.0; 18) ***	20 (14; 28)
SMA stroma density (%), *n* = 69	16 (13; 18)	15 (11; 18)	16 (12; 18)
SDF-1 stroma density (%), *n* = 67	2.9 (1.6; 4.2)	3.4 (2.3; 4.1)	3.1 (1.7; 3.9)
PDGFRβ stroma density (%), *n* = 61	6.1 (3.6; 10)	4.9 (3.8; 7.9)	9.0 (5.8; 13)
ERG endothelium density (%), *n* = 84	1.2 (0.83; 1.5)	0.83 (0.63; 1.5)	0.89 (0.66; 1.3)
**Metastases**			
Ki67 epithelium (%), *n* = 98	12 (6.0; 18)	19 (11; 35) **	18 (10; 25) *
PSA epithelium (score), *n* = 98	9 (6; 12)	4 (1; 6) ***	8 (6; 10)
AR epithelium (score), *n* = 93	9 (4; 12)	8 (3; 12)	8 (4; 12)
ERG epithelium (pos./total), *n* = 92	4/25	6/32	15/35 *
Ki67 stroma (%), *n* = 78	3.5 (2.0; 6.5)	6.0 (4.0; 8.0) *	4.0 (2.0; 7.5)
AR stroma (%), *n* = 69	2.7 (1.6; 6.0)	2 (1.3; 4.0)	3.4 (2.0; 4.0)
SMA stroma density (%), *n* = 72	4.7 (3.2; 6.8)	5.2 (4.2; 8.5)	5.3 (3.0; 6.3)
SDF-1 stroma density (%), *n* = 76	5.4 (4.1; 6.6)	4.4 (3.0; 5.6)	4.5 (3.4; 6.3)
PDGFRβ stroma density (%), *n* = 60	10 (8.3, 12)	9.6 (5.4; 13)	9.0 (6.4; 12)
ERG endothelium density (%), *n* = 82	0.87 (0.55; 1.2)	1.3 (0.95; 2.0) **	0.92 (0.80; 1.4)
MetA ^b^ (%), *n* = 70	78 (62; 86)	37 (9; 60) ***	70 (46; 84)
MetB ^b^ (%), *n* = 70	10 (1; 18)	31 (5; 65) *	14 (7; 26)
MetC ^b^ (%), *n* = 70	11 (0; 26)	11 (0; 54)	4 (0; 19)

Marker values are shown as median (25th and 75th percentiles), * *p* < 0.05, ** *p* < 0.01, *** *p* < 0.001. ^a^ A combinatory immunoreactivity score for Ki67 and PSA constructed based on the median values (13% and 8%, respectively) stratified primary tumors into 3 groups: “low Ki67, high PSA”, “high Ki67, low PSA”, and “others”. ^b^ Fractions (%) of the metastasis subtypes MetA-C were estimated based on expression levels of 157 MetA-C-associated genes, as previously described [4]. ERG, ETS-related gene; Ki67, marker for proliferation; PSA, prostate specific antigen; AR, androgen receptor; SMA, smooth muscle actin; SDF-1, stroma derived factor 1; PDGFRβ, platelet-derived growth factor receptor β.

**Table 5:** Primary tumors differentiated based on combined immunostaining of the marker for proliferation Ki67 and prostate specific antigen (PSA), in relation to the transcriptomic metastasis subtypes MetA-C of paired bone metastases.

	Ki67 Low, PSA High ^a^	Ki67 High, PSA Low ^a^	Others ^a^
	*n* = 22 (%)	*n* = 22	*n* = 26
MetA ^b^, *n* = 48	20 (91)	8 (36)	20 (77)
MetB ^b^, *n* = 11	0 (0)	8 (36)	3 (12)
MetC ^b^, *n* = 11	2 (9)	6 (27)	3 (12)

^a^ A combinatory immunoreactivity score for Ki67 and PSA constructed based on the median values (13% and 8%, respectively) stratified primary tumors into 3 groups: “low Ki67, high PSA”, “high Ki67, low PSA”, and “Others”. ^b^ Fractions of the metastasis subtypes MetA-C were estimated based on expression levels of 157 MetA-C-associated genes, as previously described [4], and each sample was classified based on its dominant MetA-C fraction.

## Data Availability

Data are not publicly available due to Swedish law, GDPR restrictions for clinical data.

## Supplementary Materials

The following supporting information can be downloaded at: https://www.mdpi.com/article/10.3390/cancers14215195/s1, Table S1: Immunoreactivity of selected epithelial and stromal markers of paired primary tumor and metastasis biopsies, sampled with less than 1.9 years in-between (below study median time), Table S2: Immunoreactivity of selected epithelial and stromal markers of paired primary tumor and metastasis biopsies, sampled with more than 1.9 years in-between (above study median time), Table S3: Correlations.
