# Supplementary material for: Epithelial and Stromal Characteristics of Primary Tumors Predict the Bone Metastatic Subtype of Prostate Cancer and Patient Survival after Androgen-Deprivation Therapy
Source: Cancers (Basel). 2022 Oct 23;14(21):5195. doi: 10.3390/cancers14215195 (PMC9659192; doi:10.3390/cancers14215195)
Supplement: Supplementary file 1 [file cancers-14-05195-s001.zip › cancers-1966124-supplementary.pdf]

Table S1. Immunoreactivity of selected epithelial and stromal markers of paired primary tumor and metastasis biopsies, sampled with less than 1.9 years in-between (below study median time)

	Primary tumor	Metastases	R_S (n)
**Epithelial markers**			
ERG (pos./total)	15/40	11/40*	0.80***
Ki67 (%), n=49	15 (10; 23)	19 (11; 26)	0.49***
PSA (score), n=49	8 (6; 9)	6 (3; 10)	0.54***
AR (score), n=44	12 (8; 12)	8 (4; 12)**	0.12
**Stromal markers**			
Ki67 (%), n=36	2.0 (1.1; 3.3)	6.0 (2.5; 7.1)***	0.33*
AR (%), n=32	17 (8.0; 25)	2.2 (1.8; 4.0)***	0.35*
SMA density (%), n=23	15 (12; 17)	5.2 (3.1; 8.4)***	0.075
SDF-1 density (%), n=25	3.3 (2.1; 4.1)	4.7 (3.6; 6.1)**	0.038
PDGFRβ density (%), n=18	6.6 (6.0; 13)	11 (8.5; 13)	0.053
ERG, endothelium density (%), n=35	1.0 (0.66; 1.5)	1.0 (0.76; 1.3)	0.012

Marker values are median and 25th and 75th percentiles, * $p < 0.05$, ** $p < 0.01$, *** $p < 0.001$. ERG, ETS-related gene; Ki67, marker for proliferation; PSA, prostate specific antigen; AR, androgen receptor; SMA, smooth muscle actin; SDF-1, stroma derived factor 1; PDGFRβ, platelet-derived growth factor receptor β.

Table S2. Immunoreactivity of selected epithelial and stromal markers of paired primary tumor and metastasis biopsies, sampled with more than 1.9 years in-between (above study median time)

	Primary tumor	Metastases	R_S (n)
**Epithelial markers**			
ERG (pos./total)	15/40	10/40	0.63***
Ki67 (%), n=49	12 (7.0; 17)	14 (7.9; 24)	0.22
PSA (score), n=49	8 (6; 12)	6 (4; 9)*	0.13
AR (score), n=48	12 (9; 12)	8 (4; 12)***	0.31*
**Stromal markers**			
Ki67 (%), n=39	1.0 (0.50; 2.3)	4.0 (3.0; 7.0)***	-0.15
AR (%), n=36	19 (13; 34)	3.1 (1.5; 5.4)***	0.19
SMA density (%), n=27	16 (13; 18)	5.8 (4.3; 7.9)***	0.072
SDF-1 density (%), n=26	3.1 (1.9; 3.9)	4.1 (2.6; 5.6)	-0.50**
PDGFRβ density (%), n=19	9.0 (3.7; 12)	9.6 (7.0; 12)	0.40
ERG, endothelium density (%), n=34	0.88 (0.66; 1.4)	1.0 (0.55; 1.4)	-0.20

Marker values are median and 25th and 75th percentiles, * $p < 0.05$, ** $p < 0.01$, *** $p < 0.001$. ERG, ETS-related gene; Ki67, marker for proliferation; PSA, prostate specific antigen; AR, androgen receptor; SMA, smooth muscle actin; SDF-1, stroma derived factor 1; PDGFRβ, platelet-derived growth factor receptor β.

Table S3. Correlations

				Primary tumor epithelium					Primary tumor stroma					
		Age diagnosis	PSA serum diagnosis	ISUP	Ki67	PSA	AR	ERG	Ki67	AR	SMA	SDF1	PDGFRB	ERG endo
Age diagnosis	Correlation Coefficient	1	0.078	0.071	-0.102	0.113	0.179	-0.015	-0.009	0.029	-0.169	0.092	-0.049	0.165
	N	97	95	97	97	97	96	85	94	96	68	66	60	83
PSA serum diagnosis	Correlation Coefficient	0.078	1	0.085	-0.179	0.199	0.187	-0.205	-0.038	0.041	-0.176	0.059	0.329*	0.213
	N	95	96	96	96	96	95	85	93	95	68	67	60	83
**Primary tumor epithelium**														
ISUP	Correlation Coefficient	0.071	0.085	1	0.224*	-0.085	0.005	-0.331**	0.226*	-0.154	-0.337**	0.211	0.122	0.261*
	N	97	96	98	98	98	97	86	95	97	69	67	61	84
Ki67	Correlation Coefficient	-0.102	-0.179	0.224*	1	-0.404***	0.09	-0.001	0.530***	-0.337***	-0.235	0.313**	-0.142	-0.002
	N	97	96	98	98	98	97	86	95	97	69	67	61	84
PSA	Correlation Coefficient	0.113	0.199	-0.085	-0.404***	1	0.096	0.001	-0.385***	0.345***	0.024	-0.008	0.163	0.203
	N	97	96	98	98	98	97	86	95	97	69	67	61	84
AR	Correlation Coefficient	0.179	0.187	0.005	0.09	0.096	1	0.084	-0.062	0.200*	-0.238*	0.199	0.068	0.122
	N	96	95	97	97	97	97	86	94	97	69	67	61	84
ERG	Correlation Coefficient	-0.015	-0.205	-0.331**	-0.001	0.001	0.084	1	-0.064	0.063	-0.109	-0.005	-0.144	-0.294**
	N	85	85	86	86	86	86	86	84	86	67	66	60	83
**Primary tumor stroma**														
Ki67	Correlation Coefficient	-0.009	-0.038	0.226*	0.530***	-0.385***	-0.062	-0.064	1	-0.277**	-0.245*	0.2	-0.339**	0.076
	N	94	93	95	95	95	94	84	95	94	67	66	59	82
AR	Correlation Coefficient	0.029	0.041	-0.154	-0.337***	0.345***	0.200*	0.063	-0.277**	1	0.06	0.013	0.232	-0.084
	N	96	95	97	97	97	97	86	94	97	69	67	61	84
SMA	Correlation Coefficient	-0.169	-0.176	-0.337**	-0.235	0.024	-0.238*	-0.109	-0.245*	0.06	1	-0.035	0.002	-0.164
	N	68	68	69	69	69	69	67	67	69	69	62	57	68
SDF1	Correlation Coefficient	0.092	0.059	0.211	0.313**	-0.008	0.199	-0.005	0.2	0.013	-0.035	1	0.153	0.312*
	N	66	67	67	67	67	67	66	66	67	62	67	54	66
PDGFRB	Correlation Coefficient	-0.049	0.329*	0.122	-0.142	0.163	0.068	-0.144	-0.339**	0.232	0.002	0.153	1	0.197
	N	60	60	61	61	61	61	60	59	61	57	54	61	61
ERG endo	Correlation Coefficient	0.165	0.213	0.261*	-0.002	0.203	0.122	-0.294**	0.076	-0.084	-0.164	0.312*	0.197	1
	N	83	83	84	84	84	84	83	82	84	68	66	61	84

		Metastasis tumor epithelium				Metastasis stroma						Metastatic subtype (%)		
		Ki67	PSA	AR	ERG	Ki67	AR	SMA	SDF1	PDGFRB	ERG endo	MetA	MetB	MetC
Age diagnosis	Correlation Coefficient	-0.075	0.082	0.083	0.002	-0.22	0.105	-0.086	0.04	0.02	-0.042	0.059	-0.118	0.165
	N	97	97	93	91	78	69	72	76	60	82	69	69	69
PSA serum diagnosis	Correlation Coefficient	-0.236*	0.354***	-0.081	-0.177	-0.15	-0.015	-0.179	-0.098	0.121	-0.073	0.365**	-0.313**	-0.068
	N	96	96	92	90	76	68	70	75	59	80	68	68	68
**Primary tumor epithelium**														
ISUP	Correlation Coefficient	-0.143	0.033	0.149	-0.397***	0.019	0.243*	-0.174	-0.168	-0.115	-0.009	0.022	-0.129	0.136
	N	98	98	93	92	78	69	72	76	60	82	70	70	70
Ki67	Correlation Coefficient	0.376***	-0.342***	0.08	-0.006	0.306**	-0.192	0.244*	-0.211	-0.086	0.294**	-0.473**	0.267*	0.15
	N	98	98	93	92	78	69	72	76	60	82	70	70	70
PSA	Correlation Coefficient	-0.235*	0.373***	0.105	0.019	-0.331**	0.113	-0.081	0.15	0.157	-0.219*	0.307**	-0.225	-0.013
	N	98	98	93	92	78	69	72	76	60	82	70	70	70
AR	Correlation Coefficient	-0.042	0.136	0.218*	0.062	-0.051	0.143	0.155	-0.215	0.017	-0.088	0.128	-0.043	0.001
	N	97	97	92	91	77	68	71	75	59	81	70	70	70
ERG	Correlation Coefficient	0.068	-0.06	-0.004	0.712***	-0.260*	-0.06	-0.071	0.051	0.067	-0.114	0.086	0.021	-0.128
	N	86	86	82	80	71	63	61	66	52	71	64	64	64
**Primary tumor stroma**														
Ki67	Correlation Coefficient	0.147	-0.154	0.082	-0.066	0.074	-0.004	0.122	-0.073	-0.145	0.359**	-0.129	0.255*	-0.214
	N	95	95	90	89	75	66	69	73	58	79	68	68	68
AR	Correlation Coefficient	-0.08	0.222*	0.089	0.098	-0.11	0.265*	-0.208	0.156	-0.066	-0.301*	0.265*	-0.248*	0.005
	N	97	97	92	91	77	68	71	75	59	81	70	70	70
SMA	Correlation Coefficient	-0.18	-0.124	-0.03	-0.09	0.08	-0.129	0.097	0.041	-0.046	0.000	-0.037	0.092	0.038
	N	69	69	65	64	56	47	50	52	40	56	51	51	51
SDF1	Correlation Coefficient	0.038	-0.016	0.167	-0.021	-0.035	-0.007	0.107	-0.268	-0.16	-0.032	-0.106	-0.012	0.08
	N	67	67	64	61	54	45	47	51	39	53	48	48	48
PDGFRB	Correlation Coefficient	0.094	0.157	0.219	0.018	-0.011	-0.113	-0.2	-0.166	0.265	-0.013	-0.095	0.027	0.277
	N	61	61	57	56	50	41	44	45	37	35	48	44	44
ERG endo	Correlation Coefficient	-0.127	0.163	0.151	-0.342**	-0.050	0.224	-0.048	-0.091	0.167	-0.094	0.110	-0.152	0.096
	N	84	84	80	79	69	61	60	63	51	69	63	63	63

		Metastasis tumor epithelium				Metastasis stroma						Metastatic subtype (%)		
		Ki67	PSA	AR	ERG	Ki67	AR	SMA	SDF1	PDGFRB	ERG endo	MetA	MetB	MetC
**Metastasis tumor epithelium**														
Ki67	Correlation Coefficient	1	-0.465***	0.149	0.169	0.538***	-0.319**	0.391***	-0.166	-0.052	0.419**	-0.394***	0.570***	-0.175
	N	98	98	93	92	78	69	72	76	60	82	70	70	70
PSA	Correlation Coefficient	-0.465***	1	0.118	-0.026	-0.335**	0.153	-0.166	0.054	0.076	-0.335**	0.367**	-0.346**	0.035
	N	98	98	93	92	78	69	72	76	60	82	70	70	70
AR	Correlation Coefficient	0.149	0.118	1	0.099	-0.028	-0.035	0.196	-0.215	0.086	-0.011	0.054	0.03	-0.044
	N	93	93	93	87	77	68	70	74	59	80	68	68	68
ERG	Correlation Coefficient	0.169	-0.026	0.099	1	-0.219	-0.058	-0.027	0.027	0.193	-0.177	0.115	-0.005	-0.094
	N	92	92	87	92	73	67	72	75	59	82	65	65	65
**Metastasis stroma**														
Ki67	Correlation Coefficient	0.538***	-0.335**	-0.028	-0.219	1	-0.245	0.503***	-0.253*	-0.059	0.409**	-0.373**	0.277*	-0.015
	N	78	78	77	73	78	62	63	63	49	70	62	62	62
AR	Correlation Coefficient	-0.319**	0.153	-0.035	-0.058	-0.245	1	-0.315*	0.144	-0.2	-0.162	0.340**	-0.400**	-0.004
	N	69	69	68	67	62	69	60	58	49	65	61	61	61
SMA	Correlation Coefficient	0.391***	-0.166	0.196	-0.027	0.503***	-0.315*	1	-0.226	0.138	0.333**	-0.373**	0.416**	-0.069
	N	72	72	70	72	63	60	72	63	53	71	58	58	58
SDF1	Correlation Coefficient	-0.166	0.054	-0.215	0.027	-0.253*	0.144	-0.226	1	0.450**	-0.210	0.223	-0.320*	0.044
	N	76	76	74	75	63	58	63	76	54	73	55	55	55
PDGFRB	Correlation Coefficient	-0.052	0.076	0.086	0.193	-0.059	-0.2	0.138	0.450***	1	-0.149	0.009	-0.018	0.093
	N	60	60	59	59	49	49	53	54	60	56	48	48	48
ERG endo	Correlation Coefficient	0.419**	-0.335**	-0.011	-0.177	0.409**	-0.162	0.333**	-0.210	-0.149	1	-0.415**	0.384**	-0.150
	N	82	82	80	82	70	65	71	73	56	82	63	63	63
**Metastatic subtype (%)**														
MetA	Correlation Coefficient	-0.394***	0.367**	0.054	0.115	-0.373**	0.340**	-0.373**	0.223	0.009	-0.415**	1	-0.518***	-0.506***
	N	70	70	68	65	62	61	58	55	48	63	70	70	70
MetB	Correlation Coefficient	0.570***	-0.346**	0.03	-0.005	0.277*	-0.400**	0.416**	-0.320*	-0.018	0.384**	-0.518***	1	-0.246*
	N	70	70	68	65	62	61	58	55	48	63	70	70	70
MetC	Correlation Coefficient	-0.175	0.035	-0.044	-0.094	-0.015	-0.004	-0.069	0.044	0.093	-0.150	-0.506***	-0.246*	1
	N	70	70	68	65	62	61	58	55	48	63	70	70	70

* $p < 0.05$, ** $p < 0.01$, *** $p < 0.001$.
